# Photorespiration: regulation and new insights on the potential role of persulfidation

**DOI:** 10.1093/jxb/erad291

**Published:** 2023-07-24

**Authors:** Angeles Aroca, Inmaculada García-Díaz, Margarita García-Calderón, Cecilia Gotor, Antonio J Márquez, Marco Betti

**Affiliations:** Instituto de Bioquímica Vegetal y Fotosíntesis (Universidad de Sevilla, Consejo Superior de Investigaciones Científicas), Américo Vespucio 49, 41092 Sevilla, Spain; Departamento de Bioquímica Vegetal y Biología Molecular, Facultad de Química, Universidad de Sevilla, C/Profesor García González, 1, 41012 Sevilla, Spain; Departamento de Bioquímica Vegetal y Biología Molecular, Facultad de Química, Universidad de Sevilla, C/Profesor García González, 1, 41012 Sevilla, Spain; Departamento de Bioquímica Vegetal y Biología Molecular, Facultad de Química, Universidad de Sevilla, C/Profesor García González, 1, 41012 Sevilla, Spain; Instituto de Bioquímica Vegetal y Fotosíntesis (Universidad de Sevilla, Consejo Superior de Investigaciones Científicas), Américo Vespucio 49, 41092 Sevilla, Spain; Departamento de Bioquímica Vegetal y Biología Molecular, Facultad de Química, Universidad de Sevilla, C/Profesor García González, 1, 41012 Sevilla, Spain; Departamento de Bioquímica Vegetal y Biología Molecular, Facultad de Química, Universidad de Sevilla, C/Profesor García González, 1, 41012 Sevilla, Spain; University of Cologne, Germany

**Keywords:** Nitrogen metabolism, photorespiration, protein persulfidation, proteomics, sulfur metabolism, transcription factors

## Abstract

Photorespiration has been considered a ‘futile’ cycle in C_3_ plants, necessary to detoxify and recycle the metabolites generated by the oxygenating activity of Rubisco. However, several reports indicate that this metabolic route plays a fundamental role in plant metabolism and constitutes a very interesting research topic. Many open questions still remain with regard to photorespiration. One of these questions is how the photorespiratory process is regulated in plants and what factors contribute to this regulation. In this review, we summarize recent advances in the regulation of the photorespiratory pathway with a special focus on the transcriptional and post-translational regulation of photorespiration and the interconnections of this process with nitrogen and sulfur metabolism. Recent findings on sulfide signaling and protein persulfidation are also described.

## Teaser

Photorespiration is a key pathway in plant metabolism. Here we review the latest insights into the regulation of this pathway and its interaction with nitrogen and sulfur metabolism.

## Introduction

**Fig. 1.**
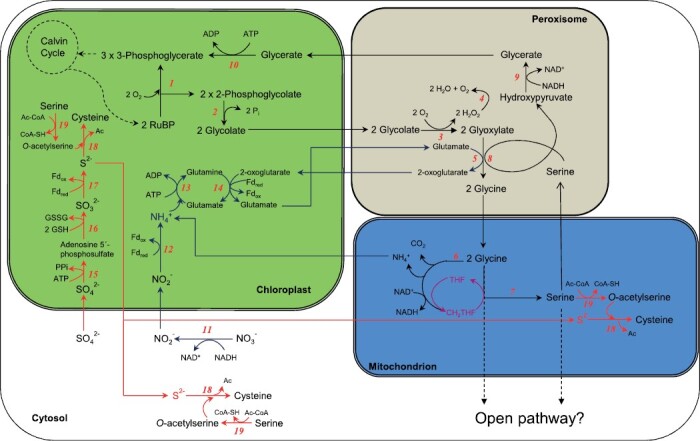
Scheme of the photorespiratory cycle and its connection with sulfur and nitrogen metabolism. (1) Rubisco; (2) PGLP, phosphoglycolate phosphatase; (3) GOX, glycolate oxidase; (4) CAT, catalase; (5) GGAT, glutamate:glyoxylate aminotransferase; (6) GDC, glycine decarboxylase complex; (7) SHMT, serine hydroxymethyltransferase; (8) SGAT, serine:glyoxylate aminotransferase; (9) HPR, hydroxypyruvate reductase; (10) GLYK, glycerate kinase; (11) NR, nitrate reductase; (12) NiR, nitrite reductase; (13) GS, glutamine synthetase; (14) GOGAT, glutamine:oxoglutarate aminotransferase; (15) ATPS, ATP sulfurylase; (16) APR, APS reductase; (17) SIR, sulfite reductase; (18) OASTL, *O*-acetylserine(thiol)lyase; (19) SERAT, serine acetyl-transferase. THF is linked to C1 units.

Life emerged >3.5 billion years ago under an anoxygenic atmosphere where ancient bacteria were able to carry out anoxygenic photosynthesis, which does not produce oxygen. Nevertheless, oxygenic photosynthesis evolved driven by ancestors of cyanobacteria, triggering a change in the atmosphere composition, enriching it in oxygen to the 21% concentration of today. Concomitantly, carbon dioxide was assimilated into biomass and, therefore, as a result of oxygenic photosynthesis expansion, the atmospheric CO_2_ concentration decreased (Lyons *et al.*, 2014). These atmospheric changes during the evolution of the Earth’s atmosphere forced the evolutionary phenomena of a new metabolic pathway intrinsically linked to photosynthesis, named photorespiration. Photorespiration therefore originated from the biochemical properties of Rubisco, the first enzyme involved in the CO_2_ fixation pathway through the Calvin–Benson–Bassham (CBB) cycle. Rubisco, in addition to its carboxylase activity, can also catalyze the oxygenation of ribulose-1,5-bisphosphate (RuBP). Due to the increase in oxygen concentration in the atmosphere, the oxygenation of RuBP by the oxygenase activity of Rubisco produces a toxic metabolite, 2-phosphoglycolate (2PG), that must be detoxified (Tolbert, 1997). This detoxification is carried out by a complex pathway, which includes several enzymatic conversions along the chloroplast, peroxisome, and mitochondria, where two 2PGs are converted to 3-phosphoglycerate (3PGA) to replenish the CBB cycle, with loss of CO_2_ and NH_3_ (Fig. 1). Photorespiration corresponds to the second most important process based on carbon flow in the terrestrial biosphere, surpassed only by photosynthesis (Bauwe *et al.*, 2012). However, photorespiration is often considered wasteful (Betti *et al.*, 2016) since it releases CO_2_ and NH_3_, and it consumes ATP and reducing power for the reassimilation of NH_3_. Consequently, during the last two decades, the greatest challenge for plant researchers has been bypassing photorespiration through different approaches, with the goal of increasing photosynthesis and consequently the yield of crops (Betti *et al.*, 2016; Fernie and Bauwe, 2020). In fact, several groups have established different ‘photorespiratory bypasses’ by introducing new metabolic pathways into the plant. Such studies have been carried out in both model plants (Eisenhut *et al.*, 2019; Cavanagh *et al.*, 2022) and crop plants, such as rice (Shen *et al.*, 2019; Wang *et al.*, 2020), where the introduction of photorespiratory bypass led to an increase in seed yield, and tobacco, where a synthetic glycolate pathway greatly increased biomass production (South *et al.*, 2019). A description of the different approaches used for bypassing photorespiration can be found in other recent works (Fernie and Bauwe, 2020; Hodges, 2022).

However, several studies have recently indicated that suppressing any photorespiratory reaction usually leads to detrimental outcomes for plants. A very large body of evidence shows that many essential processes, such as nitrogen and sulfur assimilation, depend on photorespiration, as will be described later in this review (Bloom *et al.*, 2002; Abadie and Tcherkez, 2019). Therefore, the fact that the atmospheric CO_2_ concentration is clearly predicted to increase, resulting in a decrease in the photorespiratory rate, this might threaten crop yield and food quality by reducing the protein concentration in harvests.

Plants are sessile organisms that must cope with several environmental stresses, such as heat, cold, salt, heavy metals, hypoxia, and drought. These stresses often provoke a disequilibrium in redox cellular homeostasis, increasing the intracellular reactive oxygen species (ROS) level, which might cause cellular damage, ultimately causing cell death and affecting crop yield worldwide as a result of a decrease in plant growth or disturbing fruit development. Several studies have confirmed that photorespiration is crucial for plant acclimation to several stress conditions, such as drought (Wingler *et al.*, 1999b), high light (Huang *et al.*, 2015; Z. Wang *et al.*, 2022), salinity (Ziotti *et al.*, 2019), and elevated CO_2_ (Eisenhut *et al.*, 2017). In fact, new roles of photorespiration have recently emerged beyond what was previously assumed to be a wasteful process. Therefore, photorespiration has become an important part of stress responses in plants that prevents the accumulation of ROS, even though photorespiration itself is a process that leads to ROS production (Voss *et al.*, 2013). Under abiotic stress conditions, plants not only show excess ROS production but also frequently show damage to membrane structures due to lipid peroxidation and an imbalance in ATP/NAD(P)H requirements (Voss *et al.*, 2013; Mignolet-Spruyt *et al.*, 2016). Therefore, in addition to the antioxidant defense mechanism, they have further mechanisms to protect themselves from the energy imbalance (Miller *et al.*, 2010; Golldack *et al.*, 2014; Mignolet-Spruyt *et al.*, 2016), which might directly lead to photoinhibition or photooxidation of photosystems (Walker *et al.*, 2014). Photorespiration is important in cell energetics, regenerating acceptors for primary reactions, and using reducing equivalents and ATP, thus protecting plants from photooxidation (Foyer *et al.*, 2009; Voss *et al.*, 2013).

Considering all these assumptions, it appears that photorespiration is no longer seen as a simple pathway to detoxify metabolic intermediates or recycle carbons from 2PG into 3PGA. There is an increasing amount of evidence that this pathway plays a central role in several essential metabolic functions and in the response to abiotic stress. Hence, understanding how photorespiration is regulated is a very important issue for current research. In fact, many photorespiratory enzymes have been identified as targets for several redox post-translational modifications (PTMs), and numerous studies have highlighted their sensitivity to oxidative conditions (Bartsch *et al.*, 2010). Nevertheless, other aspects of the regulatory mechanisms of photorespiration are scarce, even if the genetics and biochemistry of the photorespiratory pathway are well known (Timm and Hagemann, 2020; Hodges, 2022). Based on the importance of this metabolic pathway, especially in the context of climate change, several researchers are working to understand the dynamic regulation of photorespiration in response to environmental changes and nutrient availability. Therefore, in this review, we aim to summarize the current knowledge on the regulation of the photorespiratory pathway at the transcriptional, post-transcriptional, and post-translational levels, mostly related to the interactions of photorespiration with nitrogen and sulfur metabolism.

## Transcriptional and post-transcriptional regulation of photorespiration

Since photorespiration is intimately intertwined with photosynthesis, it is not surprising that light is an inducer of the expression of photorespiratory genes. However, there are other effectors that modulate the expression of these genes. Here, we summarize some of the main advances in the knowledge of the transcriptional and post-transcriptional regulation of photorespiration.

### Light as a regulatory signal

In addition to its role as an energy source for plants, light can also be used as a signal to trigger distinct physiological processes, including photorespiration. Similar to photosynthetic genes, genes encoding core enzymes of the photorespiratory pathway are up-regulated after exposure to light (Foyer *et al.*, 2009).

**Fig. 2.**
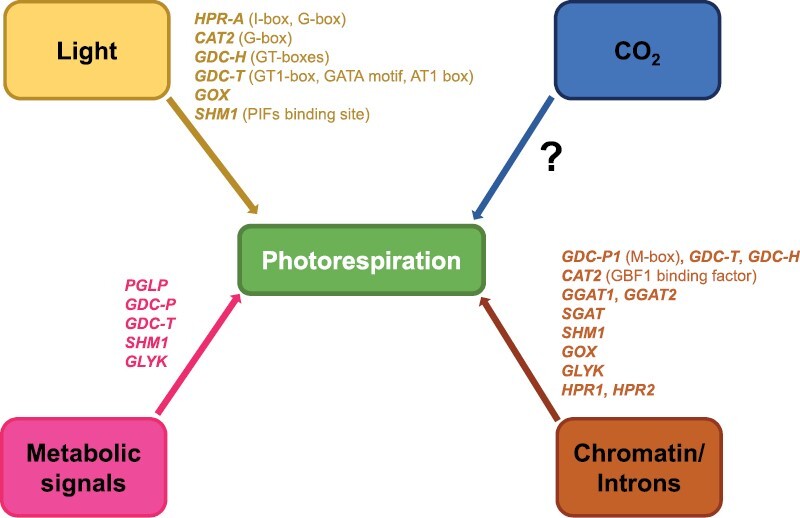
Photorespiratory genes showing transcriptional and/or post-transcriptional regulation. The genes present in the figure are hydroxypyruvate reductase (HPR-A, HPR1, HPR2); catalase (CAT2); glycine decarboxylase (GDC-H, GDC-P1, GDC-T); glycolate oxidase (GOX); serine hydroxymethyltransferase (SHM1); glutamate:glyoxylate aminotransferase (GGAT1, GGAT2); serine:glyoxylate aminotransferase (SGAT); glycerate kinase (GLYK); and phosphoglycolate phosphatase (PGLP).

The diurnal variation in the transcription of photorespiratory genes has been extensively analyzed in the model organism *Arabidopsis thaliana,* as well as in other species. Light-responsive elements (LREs) are conserved regulatory motifs located within 5ʹ-upstream regions which act as *cis*-regulatory elements involved in the control of transcription through the interaction with nuclear protein factors (Giuliano *et al.*, 1988; Gilmartin *et al.*, 1990). These LREs mediate the light induction of photorespiratory genes. For example, the l-box is present in the *HPR-A* gene of cucumber (for a list of the abbreviations of the gene names, see the legend of Fig. 2), which also contains a G-box motif (Sloan *et al.*, 1993), which was also detected in the *CAT2* gene of Arabidopsis (Laxa, 2017). GT-boxes have been identified in the *GDC-H* gene of Arabidopsis (Srinivasan and Oliver, 1995), and a GT1-binding motif is present in the *GDC-T* gene of pea (*Pisum sativum*). In this same gene, a tandem GATA motif and an AT-rich sequence equivalent to an AT-1 box have also been detected (Vauclare *et al.*, 1998). Furthermore, the presence of dark-dependent repressors such as those suggested for *HPR-A* of cucumber and *GDC-T* of pea seems to be involved (Sloan *et al.*, 1993; Srinivasan and Oliver, 1995). The tobacco *GOX* gene is also regulated by light, but indirectly, depending on the development of plastids (Barak *et al.*, 2001). It was proposed that after light exposure, a signal originating from developing chloroplasts, which is specifically perceived in the nucleus by the promoter, drives transcription (Barak *et al.*, 2001).

On the other hand, Igamberdiev *et al.* (2014) suggested that the levels of the mitochondrial photorespiratory enzymes glycine decarboxylase (GDC) and serine hydroxymethyltransferase (SHMT) on the surface of leaves, closer to the top, could be determined by gradients of light. A mechanism dependent on the phytochrome triggered by Ca^2+^ and cGMP was postulated that leads to an interaction between the active phytochrome conformation and the phytochrome-interacting basic helix–loop–helix transcription factors (PIFs), which bind to specific sequences located within the promoter, leading to higher expression of these genes where the light is more intense.

### CO_2_ as a regulatory signal

How the levels of atmospheric CO_2_ may influence the expression of photorespiratory genes has been poorly studied. Analysis of the whole transcriptome from the leaves of wild-type plants of the model legume *Lotus japonicus* grown under non-photorespiratory conditions (NPC; 0.7% v/v CO_2_) compared with active photorespiratory conditions (APC; normal air) has been carried out (Pérez-Delgado *et al.*, 2013), but no significant changes were detected in the transcript levels of photorespiratory genes. In contrast, other genes related to carbon assimilation, histones, and cell division were the most significantly modulated, as could be expected because of the differences in CO_2_ levels, which also produced differences in the growth rate of the plants. Interestingly, secondary metabolism pathways, such as the biosynthesis of flavonoids, were also modulated. Later works have further analyzed the connection between photorespiration and (iso)flavonoid biosynthesis in this plant (García-Calderón *et al.*, 2015, 2020). In agreement with recent findings of our group in *L. japonicus*, Arabidopsis plants shifted from high CO_2_ to ambient CO_2_ levels did not show significant transcriptional changes in the expression of photorespiratory genes (Eisenhut *et al.*, 2017). Altogether, these data suggest that the regulation of the photorespiratory pathway by CO_2_ tends more toward ‘quick’ regulation at the level of enzyme activities (Timm, 2020). The effect of high CO_2_ levels on plant metabolism has several layers of complexity due to the closure of stomata and high carbon content, and all these effects should be taken into consideration when performing experiments under a CO_2_-enriched atmosphere.

### Chromatin reorganization and the regulatory role of introns

The position of nucleosomes strongly influences the ability of proteins and transcription factors (TFs) to bind to DNA target sites. In Arabidopsis, the region of the *GDC-P1* promoter where an M-box is located has a low nucleosome density compared with adjacent regions, independent of diurnal regulation, suggesting an open chromatin structure that makes TF binding easier (Adwy *et al.*, 2015). The nucleosome-depleted region on the *CAT2* promoter in Arabidopsis correlates with higher gene expression, which is a direct consequence of a higher abundance of histone modifications and RNA polymerase II binding to the *CAT2* locus, hallmarks of an active gene (Laxa, 2017). Although *CAT2* is predicted to be the photorespiratory gene with the highest number of *cis*-elements in its 5ʹ-upstream region (Laxa and Fromm, 2018), the increase in H3K4me3 and H3K9ac, which allows an open nucleosome arrangement, is specifically influenced by the presence of Box1 and Box2 within the promoter, both identified as GBF1-binding factor sites (Laxa, 2017). On the other hand, the existence of sets of inverted and direct DNA repeats, such as those observed in the promoter region of *GDC-T* from pea, could lead to the formation of secondary structures that might influence the control of gene expression (Vauclare *et al.*, 1998).

Introns placed within the 5ʹ-untranslated regions (UTRs) are more abundant in the photorespiratory genes of Arabidopsis, mainly in peroxisomal genes (Laxa and Fromm, 2018). Among them is *GGAT1*, whose 5ʹUTR intron, rich in CT stretches, in addition to conferring leaf-specific expression on other exogenous promoters such as *GGAT2*, *GDC-P1*, or *GDC-P2*, is able to improve the expression through a regulation mechanism known as intron-mediated enhancement (IME), which occurs at the transcriptional level and affects the level of RNA polymerase II binding (Laxa *et al.*, 2016). Similar enhancing effects are expected to be associated with the 5ʹUTR introns of *SHM1*, *GOX*, *SGAT*, and *GLYK*, as revealed by the high IMEter scores predicted by Laxa and Fromm (2018).

A bioinformatic analysis carried out in Arabidopsis predicted that spliced transcripts could be detected for the genes *GGAT1* and *SGAT*, which encode photorespiratory enzymes (Laxa and Fromm, 2018). In the case of *GGAT2*, one of the two peroxisomal glutamate:glyoxylate aminotransferase isoforms present in Arabidopsis, there are up to four different splice forms that differ in the length of the 5ʹUTR and the position and length of the 5ʹUTR intron, although one of them seems to be the most abundant (Laxa *et al.*, 2016). The Arabidopsis *GGAT2* gene is induced at the beginning of the photoperiod, while GGAT1 is repressed (Peterhansel *et al.*, 2010). In pumpkin (*Cucurbita maxima*), it has been suggested that alternative splicing of an intron controls the subcellular localization of HPR since two HPR proteins are generated from a single pre-mRNA. *HPR1* is targeted to the peroxisome, whereas *HPR2* remains in the cytosol, and its synthesis is induced by light (Mano *et al.*, 1999).

Wiludda *et al.* (2012) proposed that inefficient splicing of an intron in the 5ʹUTR of *GDC-PA* transcripts in *Flaveria trinervia* apparently promotes RNA decay. In addition, in this same species, the phenomenon of alternative splicing was also described for the *GDC-H* gene, where two generated mRNAs, differing in the length of their coding regions, encode two isoproteins with distinct organ specificity: one predominates in roots, and the other predominates in leaves and stems (Kopriva *et al.*, 1995). Kopriva *et al.* (1996) demonstrated that this kind of alternative splicing in the gene encoding the H-subunit of GDC is a hallmark not only of *F. trinervia* but also of all advanced *Flaveria* C_4_ species. In C_4_ plants, photorespiration is restricted to the bundle sheath and is missing in the mesophyll. Most probably, the described changes in the expression and splicing of *GDC-H* transcripts are part of the tissue-specific expression of these genes and part of the stepwise evolution of C_4_ photosynthesis.

### Metabolic signals as transcriptional regulators

Several studies have shown that the overexpression or down-regulation of some genes encoding photorespiratory enzymes, such as GGAT, SGAT, or HPR, in Arabidopsis, barley, or rice transgenic plants can alter the levels of several key photorespiratory metabolites compared with wild-type plants (Wingler *et al.*, 1999a; Igarashi *et al.*, 2006; Zhang *et al.*, 2015; Modde *et al.*, 2017), including serine and glycine. Glycine acts as an inducer of several photorespiratory genes, such as *PGLP*, *GDC-P*, *GDC-T*, *SHM1*, and *GLYK* (Timm *et al.*, 2013). On the other hand, serine levels have been suggested to act as a regulatory signal (Timm *et al.*, 2013). The level of transcriptional modulation of these genes is also proportional to the concentration of serine (Timm *et al.*, 2013; Modde *et al.*, 2017). Finally, a great deal of evidence also indicates that 2PG, the first photorespiratory metabolite, communicates changes in photorespiration to other metabolic pathways (Flügel *et al.*, 2017; Timm *et al.*, 2019; Timm, 2020). A summary of the photorespiratory genes that are known to be regulated at the transcriptional and/or post-transcriptional levels is provided in Fig. 2.

## Influence of nitrogen and sulfur metabolism on the regulation of photorespiration

### Interconnections between photorespiration and nitrogen assimilation

C_3_ plants growing under NO_3_^−^ as their sole source of N showed slower growth under CO_2_ enrichment than those growing under NH_4_^+^ (Bloom *et al.*, 2002, 2012; Carlisle *et al.*, 2012). This finding was taken as an indication of the possible interconnection between photorespiration and nitrate assimilation. However, there are also other results showing that the effects of elevated CO_2_ on the nitrogen assimilation and growth of C_3_ vascular plants are similar regardless of the N form assimilated (Andrews *et al.*, 2019). Further work demonstrated that conditions that decrease photorespiration (elevated CO_2_ or low O_2_ atmospheric concentrations) inhibit NO_3_^−^ assimilation in the shoots of C_3_ plants (Bloom *et al.*, 2002; Rachmilevitch *et al.*, 2004; Bloom, 2015). In addition, the levels of absorption of nitrate nutrients and organic N accumulation levels in different plant species decreased when plants received NO_3_^−^ as the sole source of N under elevated CO_2_ conditions (Bloom *et al.*, 2010; Aranjuelo *et al.*, 2013). It is noteworthy that ^14^N and ^15^N labeling experiments showed a diminution in NO_3_^−^ assimilation under CO_2_ enrichment (Bloom *et al.*, 2010). Different explanations have been given to explain how a reduction in photorespiratory rates could inhibit NO_3_^−^ assimilation (Bloom *et al.*, 2010). The first step of primary nitrogen assimilation is the conversion of NO_3_^−^ to NO_2_^−^ in the cytoplasm of leaf mesophyll cells (Fig. 1), a process dependent on the reduced form of NAD (NADH). Photorespiration stimulates the export of malic acid from chloroplasts (Backhausen *et al.*, 1998) and increases the availability of NADH in the cytoplasm (Igamberdiev *et al.*, 2001). Therefore, it was considered that the diminution of photorespiration by elevated CO_2_ would decrease the amount of reductant available to power the reduction of NO_3_^−^. Other physiological mechanisms that may link NO_3_ assimilation and photorespiration are NO_2_^−^ translocation from the cytosol into the chloroplast and competition for reductants in the chloroplast stroma (Bloom *et al.*, 2010). As a consequence, several studies have noted that elevated CO_2_ decreases the N content of plant biomass (Rachmilevitch *et al.*, 2004; Bloom *et al.*, 2010, 2012, 2014). Other studies do not support the idea that nitrate reduction is inhibited by elevated CO_2_, pointing to a dilution of nitrogen-containing compounds by assimilated carbon at elevated CO_2_ (Krämer *et al.*, 2022). Therefore, the possible reason for the diminished nitrogen assimilation at elevated CO_2_ remains controversial. Exposure to elevated atmospheric CO_2_ has repeatedly been shown to cause an increased C/N ratio of plant biomass that could result from either increased carbon or, in relation to the acquisition of carbon, reduced nitrogen assimilation (Krämer *et al.*, 2022). Further work is still required to analyze the underlying mechanisms for the required coordination between photosynthetic carbon and nitrogen assimilation and the involvement of photorespiration. Differences in nitrate assimilation have been observed in *L. japonicus* plants depending on the external concentrations of NO_3_^−^ available for the plants. Higher or lower uptake of NO_3_^−^ was observed under APC compared with NPC in plants grown at 2 mM or 0.15 mM nitrate, respectively, indicating that high- and low-affinity NO_3_^−^ transporters behave differently in response to photorespiration (García-Calderón, 2009). However, no significant modulation or minor induction was detected in NO_3_^−^ transport or assimilatory transcripts from the transcriptomes of wild-type *L. japonicus* nitrate-grown plants when transferred from NPC to APC (Pérez-Delgado *et al.*, 2013, 2016). Further experiments are needed to compare plants grown with different nitrogen sources to study the regulation under these conditions at the transcriptional, post-transcriptional, and post-translational levels. Recent results illustrate how important changes are produced in the proteomics and C/N balance of plants under NPC versus APC (García-Calderón *et al.*, 2023).

### Interconnections between photorespiration and biological N_2_ fixation in legumes

Several studies have examined the interconnection between plant photorespiration and biological nitrogen fixation carried out by symbiotic rhizobacteria (Bloom *et al.*, 2012; García-Calderón *et al.*, 2012; Aranjuelo *et al.*, 2013). García-Calderón *et al.* (2012) analyzed this interaction using wild-type and photorespiratory mutants deficient in plastidic glutamine synthetase of the model legume *L. japonicus* grown under NPC and transferred to APC. The capacity to establish a symbiotic association with *Mesorhizobium loti* bacteria and the nitrogen fixation process were examined. The transfer of wild-type and mutant plants from high CO_2_ to air conditions affected the number and fresh weight of nodules as well as the levels of nitrogenase (measured by acetylene reduction activity), which were substantially reduced compared with the plants maintained at high CO_2_. These results indicated that photorespiration generates a negative influence on nodule formation, development, and function. Furthermore, photorespiratory mutant nodules were considerably more affected than wild-type nodules after the transfer of plants from NPC to APC. The results obtained suggested that the photorespiratory activity of the plants influences nitrogen fixation negatively through limitation of carbon flux (García-Calderón *et al.*, 2012). In studies carried out with nodulated pea plants grown under CO_2_ enrichment, enhanced whole-plant growth, increased nodule biomass, and enhancement of activities related to nodule carbon metabolism and acetylene reduction activity have been reported (Cabrerizo *et al.*, 2001).

### Interconnections between photorespiration and nitrogen assimilation (TFs and co-expression studies)

A set of gene co-expression networks was recently developed to look for specific TFs that could regulate both nitrogen metabolism and photorespiration (Pérez-Delgado *et al.*, 2016). The 30 TFs that are most connected to both nitrogen and photorespiratory metabolism according to this gene co-expression analysis are shown in Table 1. It also shows the transposon-tagged LORE1 mutant lines available in *L. japonicus* for these TFs in the regulation of photorespiration and nitrogen metabolism. We have recently isolated homozygous mutant lines in several of the genes of interest listed in Table 1, and experiments are ongoing to determine whether these TFs may play a role in the regulation of the photorespiratory cycle or in the possible coordinated regulation of photorespiration and nitrogen metabolism.

**Table 1.** List of the 30 TFs most connected to photorespiratory genes and genes for nitrogen metabolism according to gene co-expression analysis in *L. japonicus*

Gene code	TF family	Arabidopsis ortholog	No. of connections	No. of LORE1 mutant lines available
Lj3g3v1113460.1	bHLH	At2g28160.1	33	18
Lj0g3v0179799.1	WRKY	At3g58710.1	33	0
Lj1g3v0593350.1	bHLH	At1g72210.1	32	11
Lj4g3v2604440.1	bZIP	At1g72210.1	32	13
Lj4g3v3099260.1	B-BOX	At1g72210.1	32	21
Lj3g3v1631860.1	MYB-like	Ag2g37630.1	31	7
Lj2g3v1984810.1	Unknown	At4g17800.1	31	0
Lj0g3v0261399.1	Trihelix	At5g63420.1	31	114
Lj5g3v0165540.1	Zinc finger	At1g75540.1	30	15
Lj2g3v2197630.1	mTERF	At4g02990.1	30	25
Lj3g3v2517670.1	bHLH	At3g61950.1	30	15
Lj3g3v3033250.2	bHLH	At2g28160.2	27	19
Lj5g3v1412970.1	bHLH	At4g02590.1	27	13
Lj1g3v3580670.1	bHLH	At3g07340.1	26	15
Lj4g3v3055130.1	TINY	At5g25810.1	26	1
Lj3g3v0741510.1	bHLH	At3g07340.1	25	9
Lj0g3v0236339.1	MYB	At3g49690.1	25	17
Lj0g3v0350599.1	bZIP	At1g42990.1	24	4
Lj2g3v1984450.1	bHLH	At4g37850.1	23	1
Lj2g3v2771140.1	bHLH	At2g40200.1	22	7
Lj4g3v2990170.1	WRKY	At4g23550.1	22	3
Lj3g3v1631860.1	MYB	At2g37630.1	21	7
Lj4g3v0819990.1	WRKY	At2g23320.1	19	2
Lj4g3v3015070.1	Unknown	At4g12750.1	17	27
Lj5g3v1533330.1	bHLH	At1g09530.1	17	43
Lj3g3v0028580.1	bHLH	At2g22770.1	16	1
Lj2g3v1141850.1	bHLH	At1g25330.1	15	9
Lj5g3v1697630.1	bZIP	At5g10030.1	15	17
Lj4g3v0973380.1	Myb-related	At4g39250.1	15	2

The number of connections between the TF gene and the genes for nitrogen metabolism and photorespiration is reported according to the gene co-expression network generated. The number of different *L. japonicus* mutant lines available in the LORE1 database is also indicated. Data are adapted from Pérez-Delgado *et al.* (2016).

Other gene co-expression studies have suggested new clues regarding the connection of photorespiration with nitrogen compounds such as asparagine, which constitutes most of the nitrogen translocated in *L. japonicus*. Analysis of the expression of photorespiratory genes and genes for asparagine metabolism indicated that these genes show similar patterns of expression in different tissues and genotypes, pointing to a connection between asparagine metabolic genes and photorespiration (García-Calderón *et al.*, 2017). It was demonstrated that a mutant plant deficient in *LjNSE1*, a gene encoding one of the asparaginase isoforms present in *L. japonicus*, showed a dramatic decrease in the expression of the two genes encoding serine:glyoxylate aminotransferase (SGAT) (García-Calderón *et al.,* 2017). In addition, expression of the genes involved in asparagine metabolism was found to be altered in a photorespiratory mutant lacking plastidic glutamine synthase (García-Calderón *et al.*, 2017). Furthermore, it should be noted that, to date, mutants available that affect particular isoforms of asparagine synthetase or asparaginase grow well under APC and do not require NPC for growth. Further work is still required to determine whether asparagine can be used in this plant as an efficient nitrogen donor in the reactions catalyzed by glyoxylate-dependent aminotransferases, key enzymes within the photorespiratory cycle (Zhang *et al.*, 2013; Modde *et al.*, 2017; Wang *et al.*, 2019).

Co-expression analysis has also been proven to be a very promising tool for the discovery of transport proteins in photorespiration and how many different transporters, already discovered or still unknown, can integrate this pathway with carbon, nitrogen, and sulfur metabolism (Bordych *et al.*, 2013; Eisenhut *et al.*, 2015).

### Transcriptomic and metabolic changes associated with the accumulation of photorespiratory ammonium

The initial work of Keys *et al.* (1978) clearly established the existence of a photorespiratory nitrogen cycle in plants. Although photorespiration is considered a wasteful process due to the loss of CO_2_ and energy, little emphasis has been placed on the simultaneous release of NH_4_^+^ as a result of the conversion of glycine to serine. The release of NH_4_^+^ due to photorespiration has been estimated to exceed (by 10-fold) the rate of primary assimilation of NH_4_^+^ from nitrate reduction (Keys *et al.*, 1978). Based on methionine sulfoximine inhibition, it was concluded that photorespiratory ammonium is efficiently reassimilated by glutamine synthetase (GS). Although the initial thought was that cytosolic GS could be in charge of this process, the isolation of photorespiratory mutants deficient in GS enabled the demonstration that plastidic GS was the isoform responsible for efficient photorespiratory ammonium reassimilation. This particular isoform of GS was specifically lacking in photorespiratory mutants first isolated in barley (Wallsgrove *et al.*, 1987) and later also in legumes such as *L. japonicus* (Orea *et al.*, 2002). An important level of photorespiratory ammonium accumulation was observed when plastidic GS mutant plants were transferred from NPC to APC, reaching a peak at 3 d after transfer followed by a subsequent decline (Pérez-Delgado *et al.*, 2013). Concomitantly, massive transcriptomic and metabolic changes were also produced in the plastidic GS mutant plants by the onset of photorespiratory conditions, indicating that the lack of photorespiratory ammonium reassimilation has a strong influence on the regulation of gene expression in plants. In particular, coordinated repression of photorespiratory genes was shown, providing the first experimental evidence for coordinated regulation of photorespiratory genes over time (Pérez-Delgado *et al.*, 2013). Interestingly, other ammonium assimilatory enzymes, such as cytosolic GS, glutamate dehydrogenase (GDH), and asparagine synthetase (ASN), were shown to be induced under conditions of high accumulation of photorespiratory ammonium when the plastidic GS isoform is lacking (Pérez-Delgado *et al.*, 2015). In addition, the impairment of the photorespiratory cycle as a result of plastidic GS deficiency produces similar transcriptomic changes to other forms of abiotic stress, such as drought, commonly affecting other apparently unrelated pathways, such as the biosynthesis of different branches of flavonoids or isoflavonoids (García-Calderón *et al.*, 2015, 2020). It has previously been shown that plastidic GS deficiency can alter proline metabolism and the transcriptomic response under drought stress even in the absence of photorespiration (Díaz *et al.*, 2010). Considering that the GS/GOGAT pathway is the main point of connection between N and C metabolism because of the need for 2-oxoglutarate for the GOGAT reaction, the multiple links found between photorespiration and other cellular processes, including central carbon metabolism [such as the tricarboxylic acid (TCA) cycle and the γ-aminobutryic acid (GABA) shunt], amino acid metabolism (mainly glutamine, glycine, and serine), and secondary metabolism, can be easily explained (Pérez-Delgado *et al.*, 2013; Betti *et al.*, 2014; García-Calderón *et al.*, 2020). In fact, a series of co-expression studies have also confirmed the clear association between plastidic GS and carbon metabolism (Betti *et al.*, 2014). The regulatory role of pool sizes, especially of glycine, serine, glutamine, and glutamate, constitutes an interesting topic of research, as described (Leegood *et al.*, 1995; Hodges *et al.*, 2016; Timm and Hagemann, 2020). Photorespiration also has an important impact on C1 metabolism due to the methylation of tetrahydrofolate (THF) in the reaction catalyzed by GDC (Fig. 1). For a detailed review of the interaction between C1 metabolism and photorespiration, see Jardine *et al.* (2017).

### Interconnections between photorespiration and sulfur metabolism

Sulfur assimilation in plants is essential for the synthesis of cysteine, methionine, and iron–sulfur clusters, as well as for the synthesis of a wide range of cofactors and secondary metabolites that are necessary for stress responses (Feldman-Salit *et al.*, 2019). Sulfur-containing amino acids, cysteine and methionine, synthesized in plants, are essential for human and animal nutrition (Hoefgen and Nikiforova, 2008). Cysteine is synthesized from sulfide (formed from sulfate reduction) and *O*-acetyl-serine derived from serine (Fig. 1). Methionine is also closely related to serine metabolism due to its thiomethyl moiety. Its S atom is derived from cysteine and its methyl group from folates, which are involved in one-carbon metabolism with serine. Therefore, serine metabolism interconnects the metabolism of S, N, and C1 and has been shown to be involved in the development and environmental adaptation of plants (Watanabe *et al.*, 2021) (Fig. 1). Considering that serine is also involved in photorespiratory metabolism, the biosynthesis of serine can be considered an interesting interplay with unknown regulatory networks connected with sulfur metabolism, photorespiration, and many other processes in plants (Ros *et al.*, 2013). In fact, serine synthesis and its consecutive metabolism are important for the regulation of intracellular redox and energy levels and pH, particularly in stress conditions when the expression of several enzymes involved in this process is up-regulated. This makes serine a key player in the biochemical adaptation to environmental stress (Igamberdiev and Kleczkowski, 2018). Nevertheless, it is important to note that serine can be synthesized by photorespiratory and non-photorespiratory pathways (Ros *et al.*, 2014). In fact, serine formed by the glycerate and phosphorylated pathways, an alternative to photorespiration, is a precursor of glycine, while glycolate accumulates under stress conditions. These pathways can be linked to the GABA shunt via transamination reactions and via participation of the same reductase for both glyoxylate and succinic semialdehyde (Igamberdiev and Kleczkowski, 2018). Glycine can also be synthesized as a result of glyoxylate transamination in photorespiratory metabolism, and two molecules of glycine are used to produce photorespiratory serine (a precursor of cysteine) (Fig. 1). Importantly, alternative pathways to ­photorespiration for glycine biosynthesis, such as threonine aldolase, can only account for 50% of the glycine content of Arabidopsis seedlings (Joshi *et al.*, 2006), thus revealing the possible ­significance of photorespiratory glycine biosynthesis. The involvement of glycine, glycolate, and glyoxylate in photorespiratory metabolism constitutes another important point of connection between serine (and therefore S) metabolism and photorespiration. On the other hand, glycine, together with cysteine, is also required for glutathione biosynthesis, which is of crucial importance in plants and therefore represents another point of connection between photorespiration and S metabolism. Under salt stress, the increase in glutathione content has been proposed to be due to augmented photorespiratory rates, which increase the metabolic availability of glycine and serine (Herschbach *et al.*, 2010). Nitrogen assimilation has recently been shown to be integrated with photosynthetic carbon metabolism, suggesting that the metabolites glycine and serine can be diverted at significant rates from the photorespiratory pathway (Busch *et al.*, 2017). To what extent the photorespiratory pathway works as a closed cycle to generate 3PGA and as an open cycle that allows the removal of metabolites for other plant functions is a question of debate (Hodges *et al.*, 2016). However, the successful implementation of ­photorespiratory bypasses in different species (Fernie and Bauwe, 2020; Cavanagh *et al.*, 2022; Hodges, 2022) clearly indicates that there is no closed photorespiratory cycle in plants.

Compared with other aspects of the photorespiratory cycle, much less attention has been paid to the mutual influence of photorespiration and sulfur metabolism. It has been demonstrated that even a moderate impairment of photorespiration severely reduces the leaf carbohydrate status and impacts sulfur metabolism (Timm *et al.*, 2021). Abadie and Tcherkez (2019) have recently shown that S assimilation is stimulated by photorespiratory metabolism and, therefore, large photosynthetic fluxes appear to be detrimental to plant cell sulfur nutrition. On the other hand, sulfur deficiency studies have shown that a decrease in the amount of the sulfur-containing molecule *S*-adenosyl-methionine (SAM) is followed by a decrease in chlorophyll content (for which the biosynthesis of SAM is required) together with increased photorespiration (Hoefgen and Nikiforova, 2008). These factors provide a cause–effect connection with decreased photosynthesis, leading to limitations in energy assimilation, which in turn leads to a general decline in metabolism. Insufficient sulfur supply leads to its misbalance with nitrogen, which is further enforced by alterations in THF, a central cofactor in C1 metabolism that links photorespiration (Ser/Gly metabolism), sulfur assimilation (Met biosynthesis), and the dumping of disbalanced nitrogen (through enforced purine metabolism also influenced by the decreased SAM). Mutual influences between these processes form a dense network of coordination that was further assessed by integration of metabolomics and transcriptomics (Hoefgen and Nikiforova, 2008). Other authors have proved that photorespiratory mutations affecting GDC activity result in an increase in glycine and serine levels. Interestingly, the high serine levels in the GDC mutant cannot be explained by the transcript abundances of the genes of the photorespiratory pathway or by two alternative pathways for serine biosynthesis. A decline in sulfur flux into the major sulfur pools in the mutants was also observed as a result of the deregulation of genes of sulfur reduction and assimilation. It was concluded that increased serine production as a consequence of the GDC mutation deregulates the crosstalk between S, N, and C metabolism (Samuilov *et al.*, 2018). Although the sulfate assimilation pathway is tightly regulated and coordinated with the demand for reduced sulfur, little is known about the molecular mechanisms of this regulation and possible interconnections with photorespiration (Koprivova and Kopriva, 2014).

A very interesting metabolite involved in sulfur assimilation is hydrogen sulfide (H_2_S), which has always been considered very toxic for most living organisms due to its inhibitory effect on cytochrome *c* oxidase activity and therefore mitochondrial electron transport (Nicholls and Kim, 1982). Nevertheless, in the last two decades, H_2_S has emerged as a signaling molecule essential for life and is involved in different physiological and pathological processes in animals, but also in plants (Gotor *et al.*, 2019; Aroca *et al.*, 2020, 2021; Laureano-Marín *et al.*, 2020). Therefore, the biosynthesis of H_2_S is a particularly attractive topic of study concerning sulfur metabolism and photorespiration. H_2_S can originate in plants not only from sulfate reduction during the photosynthetic sulfate assimilation pathway in chloroplasts but also from different enzymatic reactions involved in cysteine metabolism. In the cytosol, l-cysteine and d-cysteine desulfhydrases generate sulfide from l- or d-cysteine, respectively. Other enzymes located not only in the cytosol but also in the chloroplast and mitochondria are NifS-like proteins, and β-cyanoalanine synthase is an enzyme that uses cysteine for the detoxification of cyanide and produces H_2_S in mitochondria (Gotor *et al.*, 2019). For a detailed scheme of the pathways that lead to H_2_S in plants, please see Gotor *et al.* (2019).

H_2_S has previously been described to positively regulate growth and physiology in plants and other photosynthetic organisms, influencing photosynthesis and photorespiration (Wei *et al.*, 2017; Cheng *et al.*, 2019; Liu *et al.*, 2021). Furthermore, a recent publication demonstrates how sulfide represses the activity of glycolate oxidase, a photorespiratory enzyme, which attenuates intracellular oxidative stress (L. Wang *et al*., 2022). Currently, it is well established that the main mechanism of action of H_2_S is through the modification of proteins by persulfidation, which involves the PTM of cysteine residues, altering the thiol group (-SH) to form a persulfide group (-SSH) (Mustafa *et al.*, 2009). Persulfidation is the main mechanism by which H_2_S regulates several physiological processes in animal and plant systems (Aroca *et al.*, 2018, 2020). Very recently, the regulatory role of persulfidation when changing photorespiratory conditions has been revealed, as described below.

## Post-translational regulation of photorespiration

Large proteomic studies have revealed that several metabolic enzymes involved in photorespiration and associated pathways, such as nitrogen assimilation or the TCA cycle, are at some point controlled by protein phosphorylation, ubiquitination, acetylation, and different redox modifications, such as methionine oxidation, thioredoxin regulation, *S*-glutathionylation (Zaffagnini *et al.*, 2012; Chardonnet *et al.*, 2015), *S*-nitrosylation (Lindermayr *et al.*, 2005; Morisse *et al.*, 2014; Hu *et al.*, 2015), or sulfenylation (Akter *et al.*, 2015; De Smet *et al.*, 2019; Huang *et al.*, 2019; Wei *et al.*, 2020), which have recently been exhaustively reviewed (Hodges, 2022), along with the consequences of these modifications in plant metabolism (Timm, 2020; Timm and Hagemann, 2020). Notably, persulfidation is another novel redox modification that has been much less studied thus far. However, in several different proteomic approaches recently performed, several proteins directly involved in photorespiration have been described as targets for this particular PTM (Aroca *et al.*, 2015, 2017; Laureano-Marín *et al.*, 2020; Jurado-Flores *et al.*, 2021; García-Calderón *et al.*, 2023). Table 2 summarizes the targets of persulfidation identified in all these proteomic approaches, classified by those isoforms involved directly in photorespiration and N and S metabolism. Interestingly, many of these photorespiratory and related proteins are also modified by other cysteine redox PTMs, as shown in Table 2, suggesting the importance of redox regulation of this pathway. In addition, persulfidation, whose role has not yet been deciphered, seems to be the most widely distributed PTM in the photorespiratory pathway. The total number of persulfidated proteins is higher than that of sulfenylated and nitrosylated proteins, which are the other two cysteine redox PTMs most widespread in the photorespiratory pathway (Table 2).

**Table 2.** Persulfidated proteins related to photorespiration and nitrogen and sulfur metabolism that are also modified by *S*-nitrosylation, *S*-sulfenylation, and *S*-glutathionylation

AGI ID	Uniprot ID	Gene ID	Name	Refs
**Photorespiration**				
AT2G13360	Q56YA5	AGT1	Serine-glyoxylate aminotransferase	P^(2,3,4)^, N^(11)^
AT4G35090	P25819	CAT2	Catalase-2	P^(1,2,3,4)^, N^(11)^
AT2G35370	P25855	GDH1	Glycine cleavage system H protein 1, mitochondrial	P^(1,2,3,4)^, N^(10)^
AT2G35120	O82179	GDH2	Glycine cleavage system H protein 2, mitochondrial	P^(2,3)^, S^(5)^, N^(11)^
AT1G23310	Q9LR30	GGAT1	Glutamate–glyoxylate aminotransferase 1 (GGT1)	P^(1,3,4)^, S^(7)^, N^(10,11)^
AT4G33010	Q94B78	GLDP1	Glycine dehydrogenase (decarboxylating) 1, mitochondrial	P^(2,3,4)^, S^(5,7)^, N^(11)^
AT2G26080	O80988	GLDP2	Glycine dehydrogenase (decarboxylating) 2, mitochondrial	P^(2,3,4)^, S^(5,7)^, G^(9)^
AT5G35630	Q43127	GLN2	Glutamine synthetase (GS2)	P^(2,3,4)^, S^(5)^, N^(10,11)^
AT3G14420	Q9LRR9	GLO1	Glycolate oxidase 1 (GOX1)	P^(1,2,3,4)^
AT3G14415	Q9LRS0	GLO2	Glycolate oxidase 2 (GOX2)	P^(1,2,3,4)^, N^(10)^
AT5G04140	Q9ZNZ7	GLU1	Ferredoxin-dependent glutamate synthase 1, chloroplastic/mitochondrial (Fd-GOGAT 1)	P^(2,3,4)^, S^(7)^
AT1G80380	Q944I4	GLYK	d-Glycerate 3-kinase chloroplastic	P^(3,4)^
AT1G68010	Q9C9W5	HPR	Glycerate dehydrogenase HPR, peroxisomal (HPR 1)	P^(2,3,4)^, N^(11)^
AT1G79870	Q9CA90	HPR2	Glyoxylate/hydroxypyruvate reductase A (HPR 2)	P^(2,3,4)^, S^(5)^
AT5G36700	P0DKC3	PGLP1A	Phosphoglycolate phosphatase 1A, chloroplastic	P^(2,4)^, N^(11)^
ATcG00490	O03042	rbcL	Ribulose bisphosphate carboxylase large chain	P^(1,2,3,4)^, S^(5,7)^, N^(10,11)^
AT1G67090	P10795	RBCS-1A	Ribulose bisphosphate carboxylase small chain 1A,	P^(1,2,3,4)^, S^(7)^, N^(10,11)^
AT5G38430	P10796	RBCS-1B	Ribulose bisphosphate carboxylase small chain 1B,	P^(3,4)^, S^(7)^, N^(10,11)^
AT5G38420	P10797	RBCS-2B	Ribulose bisphosphate carboxylase small chain 2B	P^(4)^, S^(7)^, N^(10,11)^
AT5G38410	P10798	RBCS-3B	Ribulose bisphosphate carboxylase small chain 3B,	P^(3,4)^, S^(5,7)^
AT2G39730	P10896	RCA	Rubisco activase	P^(3,4)^, S^(5,7)^, N^(10)^
AT4G37930	Q9SZJ5	SHM1	Serine hydroxymethyltransferase 1, mitochondrial (SHMT1)	P^(1,3,4),^ N^(7)^
**Other related protein isoforms**				
AT2G45630	Q56XD0	At2g45630	Putative glycerate dehydrogenase	P^(3,4)^
AT1G20630	Q96528	CAT1	Catalase-1	P^(3,4)^, S^(7)^, N^(11)^
AT1G20620	Q42547	CAT3	Catalase-3	P^(2,3,4)^, N^(11)^
AT1G11860	O65396	GDCST	Aminomethyltransferase, mitochondrial	P^(2,3,4)^, S^(5)^, N^(10,11)^
AT1G70580	Q9S7E9	GGAT2	Glutamate–glyoxylate aminotransferase 2 (GGT2)	P^(2,3)^, S^(7)^, N^(11)^
AT3G14150	Q24JJ8	GLO3	Peroxisomal (*S*)-2-hydroxyacid oxidase GLO3	P^(3)^
AT4G18360	O49506	GLO5	Glycolate oxidase 3 (GOX 3)	P^(3)^
AT2G41220	Q9T0P4	GLU2	Ferredoxin-dependent glutamate synthase 2, (Fd-GOGAT 2)	P^(2,3,4)^, S^(6,7)^
AT3G25530	Q9LSV0	GLYR1	Glyoxylate/succinic semialdehyde reductase 1	P^(3,4)^, S^(7)^, G^(9)^, N^(11)^
AT5G36790	P0DKC4	PGLP1B	Phosphoglycolate phosphatase 1B chloroplastic	P^(3,4)^
AT5G47435	Q93YQ3	PURU1	Formyltetrahydrofolate deformylase 1, mitochondrial	P^(3)^
AT5G26780	Q94C74	SHM2	Serine hydroxymethyltransferase 2 mitochondrial (SHMT2)	P^(3,4)^, N^(11)^
AT4G32520	Q94JQ3	SHM3	Serine hydroxymethyltransferase 3, chloroplastic (SHMT3)	P^(2,3,4)^, S^(6,7)^
AT4G13930	O23254	SHM4	Serine hydroxymethyltransferase 4 (SHMT4)	P^(3,4)^, S^(6,7)^
**N metabolism**				
AT1G02020	Q3E6Y8	At1g02020	Nitroreductase domain-containing protein	P^(3)^
AT5G18170	Q43314	GDH1	Glutamate dehydrogenase 1	P^(2,3)^, S^(7)^
AT5G07440	Q38946	GDH2	Glutamate dehydrogenase 2 (GDH 2)	P^(1,2,3,4)^, S^(5,7)^
AT1G32470	Q9LQL0	GDH3	Glycine cleavage system H protein 3, mitochondrial	P^(2,4)^
AT5G37600	Q56WN1	GLN1-1	Glutamine synthetase cytosolic isozyme 1-1	P^(2,3)^, S^(5)^
AT1G66200	Q8LCE1	GLN1-2	Glutamine synthetase cytosolic isozyme 1-2 (GLN1;2)	P^(2,3,4)^, N^(11)^
AT3G17820	Q9LVI8	GLN1-3	Glutamine synthetase cytosolic isozyme 1-3 (GS1)	P^(2,3)^, S^(7)^
AT5G16570	Q9FMD9	GLN1-4	Glutamine synthetase cytosolic isozyme 1-4	P^(2,3)^
AT5G53460	Q9LV03	GLT1	Glutamate synthase 1 [NADH], chloroplastic (NADH-GOGAT 1)	P^(2,3,4)^, S^(5,6,7)^, N^(11)^
AT3G03910	Q9S7A0	GSH3	Probable glutamate dehydrogenase 3 (GDH 3)	P^(3)^, S^(7)^
AT1G77760	P11832	NIA1	Nitrate reductase [NADH] 1 (NR1)	P^(2,3)^, S^(5)^
AT1G37130	P11035	NIA2	Nitrate reductase [NADH] 2 (NR2)	P^(2,3,4)^, S^(5,7)^
AT2G15620	Q39161	NIR1	Ferredoxin–nitrite reductase, chloroplastic (NiR)	P^(2,3,4)^, S^(6)^, N^(11)^
AT3G53180	F4J9A0	NodGS	Nodulin/glutamine synthase-like protein	P^(3)^, G^(9)^
AT1G08090	O82811	NRT2.1	High-affinity nitrate transporter 2.1	P^(3)^
AT5G50200	Q9FGS5	NRT3.1	High-affinity nitrate transporter 3.1	P^(3)^
**S Metabolism**				
AT2G14750	Q43295	APK1	Adenylyl-sulfate kinase 1, chloroplastic	P^(3)^, N^(11)^
AT4G39940	O49196	APK2	Adenylyl-sulfate kinase 2, chloroplastic	P^(3)^
AT4G04610	P92979	APR1	5ʹ-Adenylylsulfate reductase 1, chloroplastic	P^(3)^
AT1G62180	P92981	APR2	5ʹ-Adenylylsulfate reductase 2 chloroplastic	P^(3,4)^
AT4G21990	P92980	APR3	5ʹ-Adenylylsulfate reductase 3, chloroplastic	P^(3)^
AT3G22890	Q9LIK9	APS1	ATP sulfurylase 1 chloroplastic	P^(3,4)^, S^(6,7)^, N^(11)^
AT1G19920	Q43870	APS2	ATP sulfurylase 2	P^(3,4)^, G^(9)^
AT5G43780	Q9S7D8	APS4	ATP sulfurylase 4, chloroplastic	P^(3)^
AT4G27700	Q94A65	At4g27700	Rhodanese-like domain-containing protein 14 chloroplastic	P^(4)^, S^(5)^
AT3G61440	Q9S757	CYSC1	Bifunctional l-3-cyanoalanine synthase/cysteine synthase C1 mitochondrial	P^(3,4)^, S^(5)^, N^(11)^
AT3G04940	Q9S6Z7	CYSD1	Bifunctional l-3-cyanoalanine synthase/cysteine synthase D1 (cysteine synthase D1)	P^(3)^, N^(11)^
AT5G28020	Q9SXS7	CYSD2	Bifunctional l-3-cyanoalanine synthase/cysteine synthase D2 (cysteine synthase D2)	P^(3)^, S^(7)^
At5g28030	F4K5T2	DES1	l-Cysteine desulfhydrase 1	P^(2)^, N^(11)^
At3g62130	Q9M1R1	LC-DES	l-Cysteine desulfhydrase	P^(3)^, N^(11)^
AT4G14880	P47998	OASA1	Cysteine synthase 1	P^(3,4)^, S^(7)^, N^(11)^
AT2G43750	P47999	OASB	Cysteine synthase chloroplastic/chromoplastic	P^(3,4)^, S^(5,6,7)^, N^(11)^
AT3G59760	Q43725	OASC	Cysteine synthase mitochondrial	P^(3,4)^, S^(5,7)^
AT2G17640	Q8S895	SAT2	Serine acetyltransferase 2	P^(3)^
AT3G13110	Q39218	SAT3	Serine acetyltransferase 3, mitochondrial	P^(3)^, S^(5,7)^
AT5G56760	Q42538	SAT5	Serine acetyltransferase 5	P^(3)^
AT5G04590	Q9LZ66	SIR	Assimilatory sulfite reductase (ferredoxin) chloroplastic	P^(3,4)^, S^(6,7)^
AT1G79230	O64530	STR1	Thiosulfate/3-mercaptopyruvate sulfurtransferase 1 mitochondrial	P^(3,4)^, S^(6)^
AT3G08920	Q9SR92	STR10	Rhodanese-like domain-containing protein 10	P^(4)^
AT5G66040	Q39129	STR16	Thiosulfate sulfurtransferase 16 chloroplastic	P^(3,4)^
AT5G66170	Q9FKW8	STR18	Thiosulfate sulfurtransferase 18	P^(3)^
AT2G42220	O48529	STR9	Rhodanese-like domain-containing protein 9 chloroplastic	P^(4)^

Gene ID is given for each protein according to the UniProt database. Photorespiration includes participating isoenzymes, as illustrated by Hodges (2022). Other related protein isoforms are shown separately. P, persulfidation; N, *S*-nitrosylation; S, *S*-sulfenylation; G, *S*-glutathionylation.

^1^ Aroca *et al.* (2015); ^2^Aroca *et al.* (2017); ^3^Jurado-Flores *et al.* (2021); ^4^García-Calderón *et al.* (2023); ^5^Huang *et al.* (2019); ^6^de Smet *et al.* (2019); ^7^Wei *et al.* (2020); ^8^Hu *et al.* (2015); ^9^Dixon *et al.* (2005); ^10^Lindermayr *et al.* (2005); ^11^Hu *et al.* (2015).

All of these proteins might be regulated differently depending on the chemical environment associated with cellular oxidative stress. A recent proteomic study of peroxisomes isolated from pea plants showed that several targets of nitrosylation were involved in photorespiration and the antioxidant system (Sandalio *et al.*, 2019). In fact, the properties of cysteine residues in proteins allow a wide variety of different redox PTMs, including not only sulfenylation and persulfidation but also glutathionylation and nitrosylation. Thus, photorespiratory enzymes are modified by different PTMs, and each modification leads to a different regulation outcome for the target. For instance, the glycolate oxidase activity of pea is inhibited by *S*-nitrosylation (Ortega-Galisteo *et al.*, 2012); GDC is also negatively regulated by *S*-nitrosylation and *S*-glutathionylation (Palmieri *et al.*, 2010), and is regulated by thioredoxins (da Fonseca-Pereira *et al.*, 2020). Furthermore, thioredoxin *f* was found to redox regulate glycerate kinase in maize (Bartsch *et al.*, 2010).

**Fig. 3.**
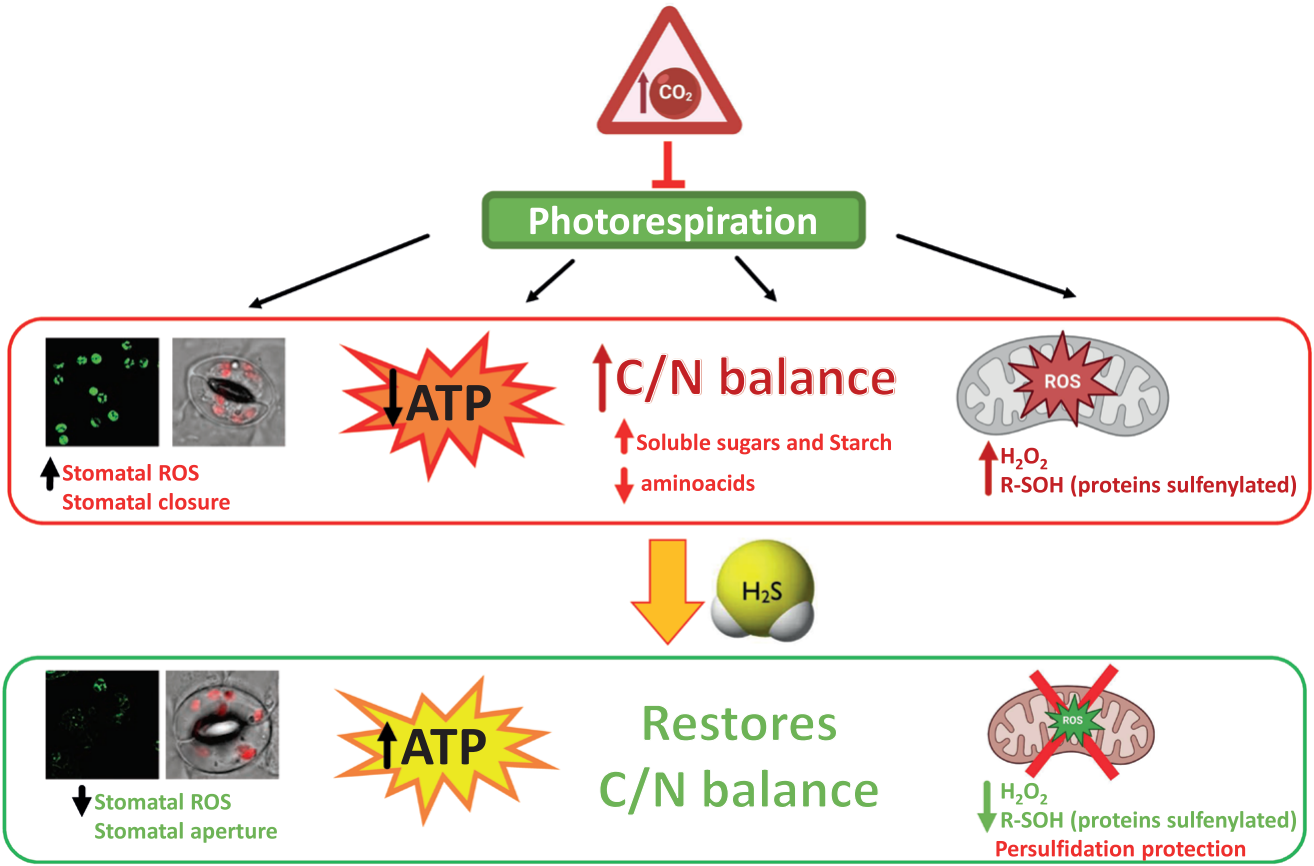
Scheme of hydrogen sulfide regulation under non-photorespiratory conditions induced by a high-CO_2_ atmosphere. More details are included in the text.

The most recent proteomic approach carried out compared the levels of persulfidation in plants grown under non-photorespiratory conditions with those of plants transferred to air. The results obtained showed a high impact on protein persulfidation levels, where 98.7% of the identified proteins were more persulfidated under suppressed photorespiration than in plants grown under air (García-Calderón *et al.*, 2023). Interestingly, redox conditions were revealed to be very different under these conditions, with a higher level of ROS detected under non-photorespiratory conditions. Given that *S*-sulfenylation is the PTM induced by hydrogen peroxide (H_2_O_2_), the crosstalk between H_2_S and H_2_O_2_ signaling was studied based on the PTMs produced by each signaling molecule. The levels of persulfidation and sulfenylation were analyzed in gel during the transition from non-photorespiratory conditions to a normal air atmosphere, and a substantial change was observed. Under conditions of suppressed photorespiration, where the H_2_O_2_ level was high, the sulfenylation levels were also higher, and, in contrast, in normal air, there was a correlation between the low H_2_O_2_ level and the high persulfidation level (García-Calderón *et al.*, 2023) (Fig. 3). The shifted persulfidation and sulfenylation waves described during the transition from non-photorespiratory growth conditions to normal air suggest the protection of sulfide against ROS species through persulfidation. These results are consistent with data previously described in mammals, where the level of protein sulfenylation decreased as persulfidation levels increased after sulfide treatment, protecting cysteines from overoxidation (Zivanovic *et al.*, 2019).

In connection with the role of sulfide in protecting against overoxidation, stomatal ROS accumulated at higher levels in plants grown under suppressed photorespiration than in plants acclimated to photorespiratory conditions. In addition, sulfide treatment induced a significant decrease in ROS accumulation in stomata, reaching similar levels to those observed in plants in normal air (García-Calderón *et al.*, 2023). These results demonstrated that sulfide regulates the ROS burst in guard cells depending on the photorespiratory conditions and therefore affects the aperture/closure of stomata (Fig. 3).

In addition, other aspects associated with photorespiratory conditions were analyzed in detail in the same study (García-Calderón *et al.*, 2023). Plants grown under suppressed photorespiration showed unbalanced carbon/nitrogen metabolism and a decrease in ATP accumulation compared with plants in normal air. However, both measurements were amended by sulfide treatment, equaling the levels in plants grown under photorespiratory conditions (Fig. 3). These results demonstrate the role of sulfide signaling under non-photorespiratory conditions through a high level of persulfidation.

**Fig. 4.**
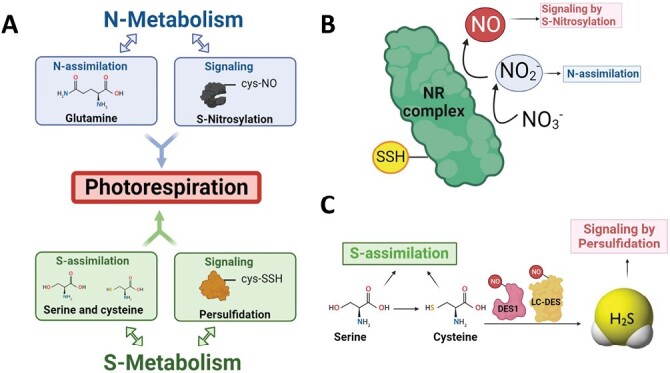
Crosstalk between N metabolism and S metabolism, both connected to photorespiration by N or S assimilation and signaling by the PTMs persulfidation and nitrosylation (A). Crosstalk between the persulfidated enzymes involved in NO generation (B) and the nitrosylated enzymes involved in H_2_S generation (C).

Therefore, photorespiratory and related proteins might be regulated differently by different PTMs with different outcomes. Further research must be performed to elucidate the relationship among these modifications and the different environmental scenarios. Concerning this topic, and connected to the focus of this review, the interconnection between nitrogen metabolism and NO signaling through nitrosylation on the one hand and sulfur metabolism and H_2_S signaling through persulfidation on the other hand deserves special attention (Fig. 4). As described above and illustrated in Fig. 1, different metabolites involved in nitrogen and sulfur metabolism play an essential role in the photorespiratory pathway, which is a known aspect of the metabolic interconnection between N metabolism and S metabolism (Fig. 4A). Additionally, the nitrate reductase complex (NR), a key enzyme in N metabolism, has been proposed to mediate NO production in plants (Chamizo-Ampudia *et al.*, 2017; Maiber *et al.*, 2022) and therefore participates in controlling the level of NO that signals through the PTM nitrosylation (Fig. 4B). On the other hand, the generation of the H_2_S signaling molecule, responsible for the PTM persulfidation, also depends on the enzymatic sources of sulfur assimilation (Fig. 4C). Interestingly, not only photorespiratory proteins but also the main proteins responsible for NO and H_2_S generation in the cytosol, such as NR, which is persulfidated, and l-cysteine desulfhydrases (LD-DES and DES1), which are nitrosylated, show some of these PTMs (Table 2; Fig. 4B, C). Therefore, the crosstalk between nitrosylation and persulfidation in the context of the photorespiratory pathway and its interconnection with N and S assimilation are very interesting aspects for future studies.

## Future perspectives

As summarized in this review, photorespiration stands at the crossroads of several primary metabolic pathways and plays a key role in the response to different types of stress. While the basic genetics and biochemistry of photorespiration are well known, there are still several open questions regarding the shuffling of photorespiratory metabolites between organelles, the regulation of the pathway, and the necessity of a high photorespiratory flux for the assimilation of several key nutrients, especially N and S. We have shown how photorespiration is regulated at the transcriptional, post-transcriptional, and post-translational levels. Recent advances hint at possible roles for TFs in the regulation of photorespiration. Mutants available in these TFs would help determine whether they can regulate photorespiratory gene expression independently or in a coordinated manner with nitrogen, sulfur, and other related metabolic pathways (carbon and secondary metabolism) and stress responses in the plants. The possible role of sulfide signaling and the modification of cysteine residues through persulfidation and its crosstalk with other cysteine redox PTMs for the regulation of this ancient metabolic pathway also constitutes a very novel and interesting topic of research. Understanding the crosstalk between the different PTMs of the photorespiratory enzymes and related metabolism and how they affect the enzyme activities would also be of great importance in the near future, which would help in the design of tools to regulate photorespiration and therefore to improve the resistance of plants to climatic change.
